# Bursting at the Seams

**DOI:** 10.1093/cid/ciaf488

**Published:** 2026-02-25

**Authors:** Maxwell Olenski, Flora Poon, Callum Maggs, Sheree Poulton, Amy Crowe, Jonathan Darby

**Affiliations:** Infectious Diseases Department, St Vincent's Hospital, Fitzroy, Victoria, Australia; Microbiology Department, St Vincent's Hospital, Fitzroy, Victoria, Australia; School of Translational Medicine, Monash University, Melbourne, Victoria, Australia; Dermatology Department, St Vincent's Hospital, Fitzroy, Victoria, Australia; Infectious Diseases Department, St Vincent's Hospital, Fitzroy, Victoria, Australia; Microbiology Department, St Vincent's Hospital, Fitzroy, Victoria, Australia; Immunopathology Department, Royal Children's Hospital, Parkville, Victoria, Australia; Infectious Diseases Department, St Vincent's Hospital, Fitzroy, Victoria, Australia; Microbiology Department, St Vincent's Hospital, Fitzroy, Victoria, Australia; Infectious Diseases Department, St Vincent's Hospital, Fitzroy, Victoria, Australia

## QUESTION

**Figure 1.**
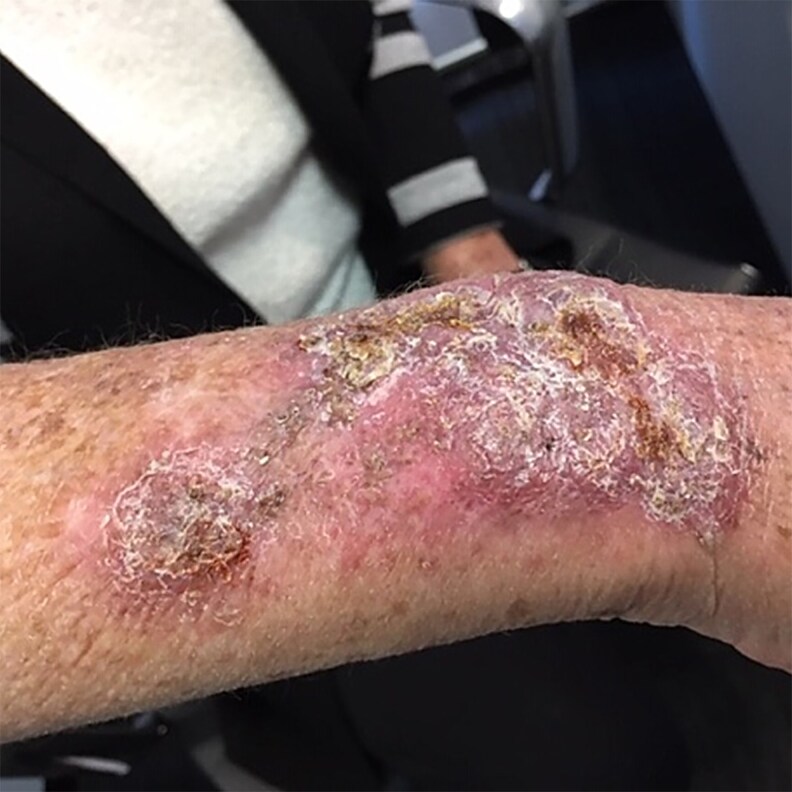
Right distal forearm violaceous, crusted plaque.

A 74-year-old female was referred from the Dermatology Clinic with a 12-month history of a slowly progressive, infiltrated, violaceous, crusted plaque approximately 12 × 4 cm located over the extensor surface of the right forearm (Figure 1). The lesion occurred with insidious onset without preceding trauma and was not associated with systemic features; she has no previous history of similar lesions or recurrent skin and soft tissue infections. It began as 3 discrete papules before coalescing and was refractory to topical therapies and, following biopsy, extended with scaling and suppuration.

Her past medical history was notable for a putative diagnosis of pulmonary sarcoidosis following a protracted respiratory illness nearly a decade prior, with chest imaging demonstrating diffuse ground-glass changes and extensive reticulonodular appearance, alongside hilar and mediastinal adenopathy. Open-lung biopsy subsequently demonstrated granulomatous pneumonitis. The patient was monitored clinically thereafter, and did not commence immunosuppressive therapy.

The patient was Australian-born and lived on a 40-acre rural property where she cultivated native plants. Further history revealed that her son had died in his teens due to complications of X-linked chronic granulomatous disease (CGD), at which point the patient and her daughter had undergone simultaneous testing, which indicated carrier status. No genetic testing was available at that juncture, or subsequently pursued. Routine blood tests revealed normal inflammatory markers and biochemistry, including normal angiotensin converting enzyme (ACE) levels.

What is your diagnosis?

Diagnosis: cutaneous *Purpureocillium lilacinum* heralding a diagnosis of late-onset chronic granulomatous disease due to skewed X-inactivation

**Figure 2.**
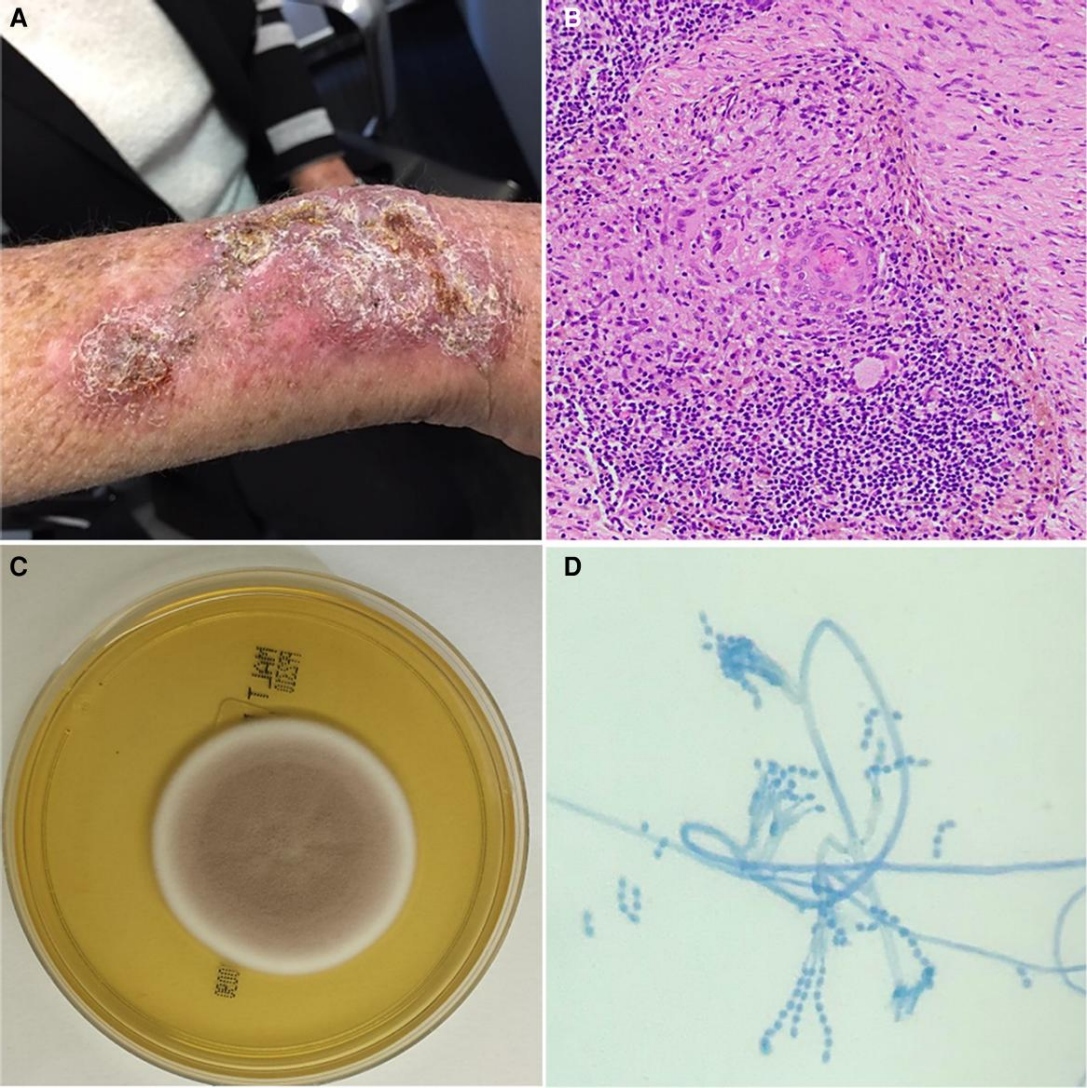
*A*, Right distal forearm violaceous, crusted plaque. *B*, Histopathological examination shows focal non-necrotizing granulomata, alongside chronic superficial inflammation including surrounding a mid-dermal blood vessel (original magnification ×200). *C*, Fungal culture on malt extract agar at 25°C showing suede-like, vinaceous colonies. *D*, Lactophenol cotton blue staining showing characteristic phialides swollen at their bases, alongside fusiform conidia produced in divergent chains from rough-walled conidiophore stipes.

Histopathology of the right forearm lesion revealed focal non-necrotizing granulomata with chronic inflammation (Figure 2*B*). Microscopy revealed numerous polymorphonuclear cells, and no pathogens were isolated on routine culture media. However, fungal culture on malt extract agar yielded fast-growing colonies, which were suede-like and vinaceous in color (Figure 2*C*). Lactophenol cotton blue staining revealed phialides that were swollen at their bases, alongside fusiform conidia produced in divergent chains (Figure 2*D*). The isolate was subsequently identified as *Purpureocilium lilacinum,* which was confirmed on DNA sequencing of the internal transcribed spacer region of the large ribosomal subunit.

**Figure 3.**
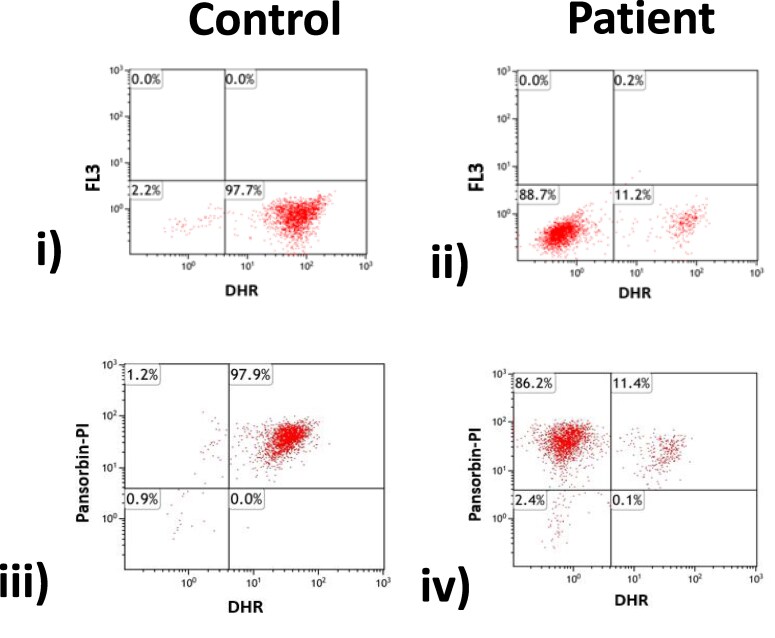
Dihydrorhodamine (DHR) flow cytometry assay in a healthy control (left) and patient (right). To assess neutrophil superoxide production, whole blood is incubated with phorbol myristate acetate (PMA; i and ii) or propidium iodide (PI-)–labeled Pansorbin, Merck, Germany (heat-killed *Staphylococcus aureus*; iii and iv). The patient demonstrates 2 populations of granulocytes, with only 11% undergoing oxidative burst in response to both PMA and Pansorbin, compared with 98% undergoing oxidative burst in the healthy control. The dual neutrophil populations suggest that the patient is a carrier of X-linked chronic granulomatous disease. The FL3 axis represents fluorescence intensity detected in the FL3 channel (a red-emission fluorochrome), whereas the Pansorbin-PI axis reflects cellular viability.

*Purpureocilium lilacinum,* formerly known as *Paecilomyces lilacinus,* is a ubiquitous hyaline hyphomycete frequently found in soil and decaying vegetation. It is a rare cause of cutaneous infection, although, if present, is frequently associated with immunocompromised states with a predilection for CGD [1]. Contemporary repeat neutrophil function testing using the dihydrorhodamine reduction (DHR) assay revealed that only 11% of granulocytes underwent oxidative burst, with a characteristic mosaic pattern of decreased oxidase activity seen in X-linked carriers of CGD (Figure 3).

Chronic granulomatous disease is a primary immunodeficiency affecting the phagocytes of the innate immune system. The disease is characterized by the increased susceptibility to catalase-positive bacterial and fungal infections caused by mutations in any of the 4 genes encoding subunits of nicotinamide adenine dinucleotide phosphate (NADPH) oxidase, resulting in decreased or absent microbicidal superoxide-generating activity [2]. Chronic granulomatous disease occurs in 1 in every 200 000 live births in the United States, with nearly identical incidence rates across racial and ethnic groups [3]. Despite this, there are few data on global trends. The most common cause of CGD is a defect in the Cytochrome B-245 Beta Chain gene (*gp91^phox^*), located on the short arm of the X chromosome [4]. There is a modest male preponderance due to a predominance of X-linked disease, wherein approximately one-third of mutations are de novo. Cutaneous manifestations of CGD occur in 60%–70% of cases and encompass a variety of infective and inflammatory lesions [5].

A strong relationship has been established between skewed inactivation of the wild-type allele and infection risk; a low DHR+ percentage strongly predicts infection risk in X-linked carriers, and carrier state itself is associated with autoimmune manifestations including photosensitive skin rashes, aphthous ulcers, inflammatory arthopathies, and inflammatory bowel disease [2]. Moreover, females with less than 20% of normal oxidase activity can manifest a severe CGD phenotype mimicking that of X-linked disease [4]. The onset of clinical phenotypes, including infective presentations, is not well described [6].

Similarly, the optimal treatment for *P lilacinum* has not yet been established, including choice and duration of therapies. Despite this, the novel triazoles tend to have low minimum inhibitory concentrations in vitro [7, 8], although—as with other rarely encountered pathogens—no interpretive breakpoints exist for the antifungals tested [9].

The patient commenced a course of mold-active triazole antifungals with regular clinical review and eventual cessation of antifungal therapy following resolution of her lesion at 12 weeks. Future treatment considerations revolve around antifungal and antibacterial prophylaxis, as well as immunomodulation and vaccination to curtail morbidity [10]. Further studies are necessary to define the roles of stem cell transplantation and gene therapy in X-linked carriers, as the degree of lyonization can prove dynamic [3, 4, 10].

Although genetic studies were not pursued, on balance—given the clinical presentation, affected male offspring, and clinical history of granulomatous pneumonitis—we surmise that the patient was now manifesting a forme fruste clinical CGD phenotype due to skewed X-inactivation [4]. Indeed, subsequent detailed review of the historic lung biopsies and pulmonary radiology—together with normal serum ACE levels—serves to challenge the notion of pulmonary sarcoidosis.

This case highlights the possible emergence of CGD phenotypes among X-linked carriers, including infective and autoimmune manifestations. Education on carrier status, institution of preventative measures (including protective clothing, modified behaviors to prevent skin trauma, and prophylactic therapeutics), in addition to early recognition of infection are paramount for prompt pathogen-directed therapy.
